# Wrinkly-Spreader Fitness in the Two-Dimensional Agar Plate Microcosm: Maladaptation, Compensation and Ecological Success

**DOI:** 10.1371/journal.pone.0000740

**Published:** 2007-08-15

**Authors:** Andrew J. Spiers

**Affiliations:** Department of Plant Sciences, University of Oxford, Oxford, United Kingdom; Oxford University, United Kingdom

## Abstract

Bacterial adaptation to new environments often leads to the establishment of new genotypes with significantly altered phenotypes. In the Wrinkly Spreader (WS), ecological success in static liquid microcosms was through the rapid colonisation of the air-liquid interface by the production of a cellulose-based biofilm. Rapid surface spreading was also seen on agar plates, but in this two-dimensional environment the WS appears maladapted and rapidly reverts to the ancestral smooth (SM)-like colony genotype. In this work, the fitness of WS relative to SM in mixed colonies was found to be low, confirming the WS instability on agar plates. By examining defined WS mutants, the maladaptive characteristic was found to be the expression of cellulose. SM-like revertants had a higher growth rate than WS and no longer expressed significant amounts of cellulose, further confirming that the expression of this high-cost polymer was the basis of maladaptation and the target of compensatory mutation in developing colonies. However, examination of the fate of WS-founded populations in either multiple-colony or single mega-colony agar plate microcosms demonstrated that the loss of WS lineages could be reduced under conditions in which the rapid spreading colony phenotype could dominate nutrient and oxygen access more effectively than competing SM/SM-like genotypes. WS-like isolates recovered from such populations showed increased WS phenotype stability as well as changes in the degree of colony spreading, confirming that the WS was adapting to the two-dimensional agar plate microcosm.

## Introduction

**Figure 1:**
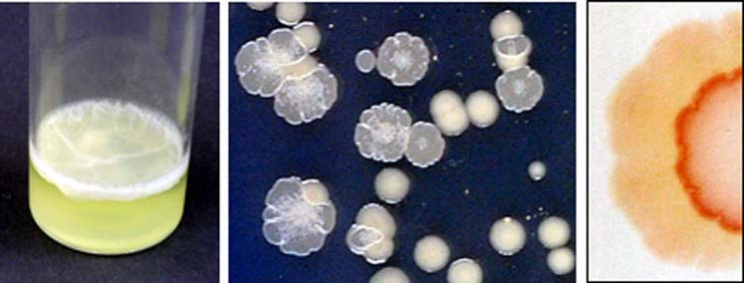
The Wrinkly Spreader (WS) produces biofilms at the air-liquid interface and wrinkled colonies on agar plates. The ecological success of the WS in static 6ml liquid King's B (KB) microcosms is the ability to produce a substantial (3.8 cm^2^, 1–1.5 mm depth) biofilm at the air-liquid interface (left). The WS genotype produces a wrinkled colony morphology on KB agar plates in contrast to the smooth colonies of the wild-type *P. fluorescens* SBW25 strain (middle). After a long period of incubation, SM-like revertants appear at the edge of the WS colony (right). On KB plates containing Congo Red, the central WS-like portion of the colony binds the dye and has a strongly-coloured red rim whilst the SM-like outer ring is pale and does not bind significant amounts of dye.

The bacterium *Pseudomonas fluorescens* SBW25 has been used extensively to examine adaptive radiation and evolution in experimental microcosms, where populations rapidly diversify to occupy different ecological niches offered by static liquid cultures [**1**]. Of these, the colonisation of the air-liquid (A-L) interface by the wrinkly-spreader (WS) genotype is the most spectacular (**Figure 1**), leading to the development of a substantial cellulose matrix-based biofilm. This rapid two-dimensional spreading is also seen in WS colony growth, where the wrinkled colonies are in contrast to the smooth (SM) rounded colonies of the ancestral non-biofilm forming wild-type strain, and requires cellulose and attachment-factor expression [**2**], [3], [**4**].

In the two-dimensional microcosm presented by the agar plate, WS might be expected to out-compete SM-like genotypes by rapidly colonising surface area and thus dominating both nutrient and oxygen access. However, WS is remarkably maladapted to growth on King's B (KB) [**5**] and Luria-Bertani (LB) [**6**] plates, and SM-like revertants (SM-LR) rapidly arise, implying that further adaptation of the WS has occurred. Just as the nutrient-rich KB liquid microcosm was a novel environment requiring adaptation by SBW25 originally isolated from the Sugar beet rhizosphere [**7**], [**8**], the two-dimensional agar plate is another, presenting new opportunities for bacterial surface colonisation. Although the rapid spread across the surface to control resources appears an obvious adaptive advantage, the development of chemical gradients (of nutrients, metabolites, O_2_, pH, *etc.*, e.g. [**9**], [**10**]) by developing colonies suggest that competitive interactions at the local neighbourhood level may be just as important for success in this environment. Bacterial colony development involves complex interactions and behaviours at the individual and population levels, responding to a variety of physical and biotic signals ([**11**], [12], [**13**] and references therein). Mutations also affect colony morphology, producing clonal sectors arising from a single mutation, and circular rings produced by independent mutations occurring in the same colony expansion phase which result in multiple clones with a similar phenotype ([**14**] and references therein). However, phenotypic changes have often carried the negative connotation of genomic instability in microbiology, and the loss of pathogenicity factors, virulence and ecological success is a consequence of prolonged maintenance using *in vitro* media and model hosts [**15**]. Nevertheless, adaptation to novel environments using experimental microcosms may be used to uncover important aspects of adaptation, diversification and evolution [**16]**, [17], [**18**].

In the particular instance of the WS on KB agar plates, the basis of the WS maladaptation is not understood. It may be the result of the primary WS phenotype (the over-expression of cellulose and attachment factor) or a non-WS–specific consequence of the original *wspF*S301R mutation [**19**]. This mutation results in the constitutive activation of the cyclic-*di*-GMP–associated, GGDEF domain-containing response regulator, WspR, which in turn leads to the over-expression of cellulose and attachment factor [**2**], [3], [**19**]. In *P. aeruginosa* PA01, WspR is involved in the regulation of cell aggregation and colony morphology (though PA01 lacks cellulose synthase genes) [**20**], and more widely, cylic-*di*-GMP/GGDEF proteins are involved in aspects of bacterial surface colonisation [**21]**, [**22**]. Interestingly, expression of the constitutively-active WspR19 (R129C) mutant protein *in trans* in a number of different *Pseudomonas* spp. activates cellulose expression, biofilm formation and sometimes also the expression of a WS-like colony morphology [**23**]. Proteomics comparison of the WS and SM suggest that the expression of at least 46 proteins are altered by *wspF*S301R, none of which appear to be involved in the primary WS phenotype [**24**]. Furthermore, metabolic differences reported amongst independent WS isolates [**25**] suggest negative pleiotropic effects associated with the WS phenotype.

In this work, WS stability and reversion are used to investigate the nature of the poor adaptation (maladaptation) of the WS to the two-dimensional agar plate microcosm. Using defined WS mutants, the maladaptive characteristic of the WS phenotype is identified and shown to be a target for further adaptation. Extended incubation of WS populations on the two-dimensional agar plate microcosm indicates that the WS enjoys limited ecological success under conditions where colony surface expansion is possible, and that WS-like genotypes can evolve showing altered colony characteristics and greater stability. The progress of the ancestral WS to SM-LR and hardy WS-lineages is a further demonstration of the adaptive powers and ecological success of microorganisms such as *P. fluorescens* SBW25.

## Results and Discussion

### The WS is unstable and has a low fitness in the two-dimensional agar plate microcosm

**Figure 2:**
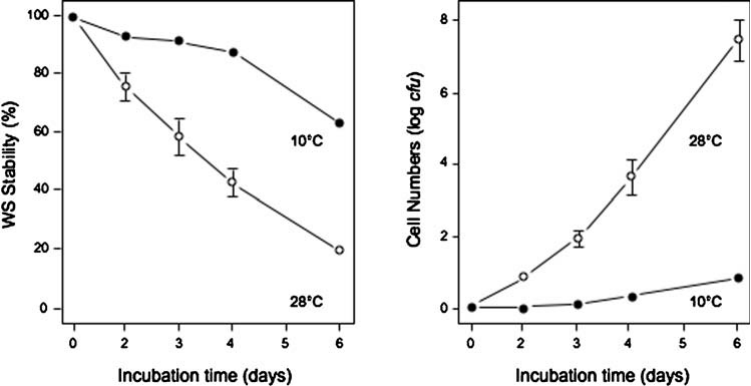
The degree of WS reversion on KB agar plates was higher when incubated at 28°C than at 10°C. The percentage of cells still showing the WS phenotype (WS Stability) recovered from colonies incubated for varying periods (left) and the total cell numbers (right) are indicated. Note that WS stability (the retention of the WS genotype) is the inverse of WS reversion to SM-LR (the loss of the WS genotype). Colonies were incubated on KB plates at 28°C for 3-days before sampling. Mean±SE indicated.

Continued incubation of the maladapted WS on KB plates drives the further adaptation of this genotype, producing SM-like revertants (SM-LR) that rapidly dominate the remaining WS lineages within the colony. These revertants did not appear to express significant amounts of cellulose as assessed by colony staining on Congo Red plates [**2]**, [**3**], further differentiating them from the ancestral WS that produced heavily-stained colonies. The appearance of SM-LR was apparent early in WS colony development when incubated at 28°C and retarded in colonies maintained at 10°C, suggesting a growth rate-dependency (the average minimal number of generations in 6-days at 10°C and 28°C was calculated from the raw *cfu* data to be ∼11 and 14, respectively) (**Figure 2**). WS stability was also found to be sensitive to level of nutrients, with a 2.2× higher WS stability on KB plates containing 0.1× proteose peptone (2 g/L) than on standard KB plates (20 g/L) at 28°C after 3-days (%WS: 0.1×, 61.8±1.8%; 1×, 28.2±8.3%; Welch ANOVA, *F*_1,2.1836_ = 15.7127, *P* = 0.0502).

The domination of WS by SM-LR genotypes within colonies suggested that SM-LR had a faster growth rate than the WS. This was confirmed using six independent SM-LR isolated from 3-day old WS colonies, and the mean relative growth rate of the SM-LR colonies on KB plates was found to be significantly greater that the WS growth rate (Relative growth over 24 hr: WS, 1×; SM-LR, 1.4–23.3×; T-Test, *t*_5_ = 2.1772, *P* = 0.0407).

**Figure 3:**
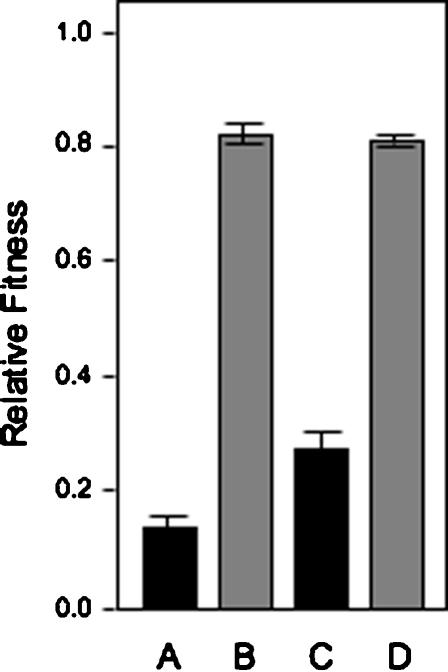
The relative fitness (W) of the WS compared to SM/SM-LR in mixed colonies is sensitive to population density and less sensitive to nutrient levels. Shown are W values for WS in mixed WS/SM/SM-LR colonies grown on normal KB plates inoculated at high (A) and low (B) cell densities, and for mixed colonies grown on plates containing 0.1× proteose peptone inoculated at high (C) and low (D) cell densities. (A) and (B) are significantly different to (C) and (D) (Tukey-Kramer HSD, *q* = 3.46172, α = 0.05). The inoculum consisted of a 40∶60 WS∶SM mixture at 10^6^ *cfu* (high density, 1×) and 10^3^ *cfu* (low density, i.e. 0.001×) applied as a 5 µl drop to the centre of the plate. Note that SM-LR lineages arise from WS cells, producing a mixed colony of WS, SM and SM-LR cells; W of WS is therefore the relative fitness of WS compared to (SM plus SM-LR). W values were determined after 3-days incubation at 28°C. Mean±SE indicated.

In order to determine the relative fitness (W) of the WS within a mixed colony, WS and SM were competed within single colonies for 3-days at 28°C. W of WS was determined at two different cell densities on 0.1× proteose peptone and standard KB plates (**Figure 3**). Two-way ANOVA indicated that the initial cell densities had a significant effect on W (*F*_1,6_ = 180, *P*<0.0001) with the higher W values found for the lower cell density, but that proteose peptone had no significant effect (*F*_1,6_ = 2, *P* = 0.207), nor was there a significant interaction effect (*F*_1,6_ = 2, *P* = 0.207). The range of W determined for WS in colonies (0.13±0.05–0.83±0.04) can be compared to the fitness (0.33) of the WS compared to the SM in shaken KB liquid microcosms in which there was no adaptive advantage of biofilm-formation [**2**], and the fitness range (−0.2–0.8) of WS genotypes that had accumulated catabolic defects over a period of 100 generations compared to reference WS isolates [**25**].

### Cellulose expression is the target for WS adaptation to the two-dimensional agar plate microcosm

The finding that SM-LR did not express significant levels of cellulose suggested that the over-expression of cellulose was the primary maladaptive character of the WS phenotype on KB plates. This could be examined by comparing the WS reversion rates occurring within the SM-like colonies of WS-4, WS-12 and WS-13, where high reversion levels could be associated with the expression of different components of the WS phenotype (Table 1). WS-4 and WS-13 are phenotypicaly SM, but express the WS colony morphology when complemented *in trans* by pVSP61-WspR19 and pCSA, respectively [**2]**, [**4**], [**26**]. In contrast, WS-12 is SM on KB plates but is WS on LB plates (the molecular basis of this remains unknown) [**2**]. When these mutants were assessed for reversion after 3-days incubation on KB plates at 28°C, no SM-LR were detected except when WS-12 was incubated at 28°C on KB plates (%WS: WS-4, WS-13 & WS-12 on LB, 100%; WS-12 on KB, 67%; cf. WS, 28%), indicating that the maladaptive WS character on agar plates must be the over-expression of cellulose.

**Table 1:** Reversion rates (instability) in WS mutants can identify the nature of the maladaptive characteristic.

Maladaptive character	Reversion (instability) expected/possible	No reversion expected
Expression of attachment factor^a^^,^ b	WS•, WS-12/LB• & WS-13	WS-4 & WS-12/KB
Expression of cellulose^a^^,^ b	WS• & WS-12/LB•	WS-4, WS-12/KB & WS-13
Pleiotropic effect of WspR activation^b^	WS•, WS-12/KB, WS-12/LB• & WS-13	WS-4
Additional consequence of the *wspF* mutation	WS•, WS-4, WS-12/KB, WS-12/LB• & WS-13	

a Requires WspR activation.

b Requires *wspF* mutation.

• Reversion (instability) seen in these cases.

WS-12/KB and WS-12/LB refer to WS-12 grown on KB and LB plates, respectively.

Compensatory mutations in SM-LR reducing the impact of the maladaptive characteristic of the WS phenotype would therefore be expected to reduce or stop cellulose expression entirely. This could occur via the intra-genetic reversion of the WS *wspF*S301R mutation back to the SM (wild-type) sequence, or via inter-genetic reversion at other loci involved in the regulation or expression of cellulose. The complete sequence of *wspF* was obtained for each of five independent SM-LR isolates and all were found to be identical to the WS *wspF*S301R sequence, suggesting that intra-genetic reversion was unlikely to be the means of genetic reversion in these populations. Re-streaking of SM-LR isolates from 3-day WS colonies on Congo Red plates [**2]**, [**3**] suggest that these revertants no longer express significant amounts of cellulose and/or attachment factor. This was further confirmed by the qualitative examination of 12 SM-LR (including those mentioned previously) by Calcofluor fluorescent microscopy [**3]**, [**4**]. Seven of these isolates did not appear to express significant amounts of cellulose (i.e. were SM-like) and the remaining five showed reduced levels of cellulose expression when compared to the WS (whilst the minimum amount of cellulose expression required to maintain a WS colony morphology is not known, expression of WS-levels of cellulose by SM is not sufficient to produce a WS-like colony morphology [**2**]).

### Cellulose over-expression by the WS diverts energy away from cell growth

The expression of cellulose (a β-[1]-[4]-linked polymer of glucose) must have a significant impact on the partitioning and utilisation of energy available to WS cells. WS stability was found to be very similar on 25 mM glucose (*Gluc*) and 40 mM succinate (*Suc*) minimal plates at 28°C (%WS: *Gluc*, 28.5±6.5%; *Suc*, 20.2±13.5%, Welch ANOVA, *F*_1,2.8833_ = 0.3066, *P* = 0.6198), indicating that the WS maladaptive pressure to revert to SM-LR was similar when utilising either carbon source. Increasing the amount of glucose resulted in lower WS stability, but when succinate was added to glucose plates instead, stability was significantly increased (%WS: 50 mM *Gluc*, 18.0±7.1; 25 mM *Gluc* plus 40 mM *Suc*, 52.6±8.4; Welch ANOVA, *F*_1,3.8785_ = 9.8613, *P* = 0.0363). This suggests that succinate could be used to replenish energy reserves normally reduced by the diversion of energy into cellulose production. Whilst glucose can be diverted immediately for cellulose production, glucolysis is necessary to provide energy for cellular growth (via the TCA cycle through pyruvate and acetyl-CoA; see www.genome.jp/kegg/pathway.html for a schematic of the glycolysis/gluconeogenesis reference pathway). In contrast, succinate provides energy first for cellular growth (i.e. it enters the TCA cycle directly) and then for gluconeogenesis. The amount of energy diverted into cellulose had a significant impact on growth rates, as the WS was found to have a lower average growth rate than WS-13 on KB plates (Relative growth over 24 hr: WS: 1.0×; WS-13: 1.43×; T-Test, *t*_2_ = −2.4291 *P* = 0.0679) (the change in growth rate must be due to the expression of cellulose rather than the transcription/translation of the *wss* genes, as there was no difference in the growth rate of SM and SM-13 containing the same mini-Tn*5* insertion as WS-13 [**2**]).

### WS-like genotypes can adapt in the two-dimensional agar plate microcosm

**Figure 4:**
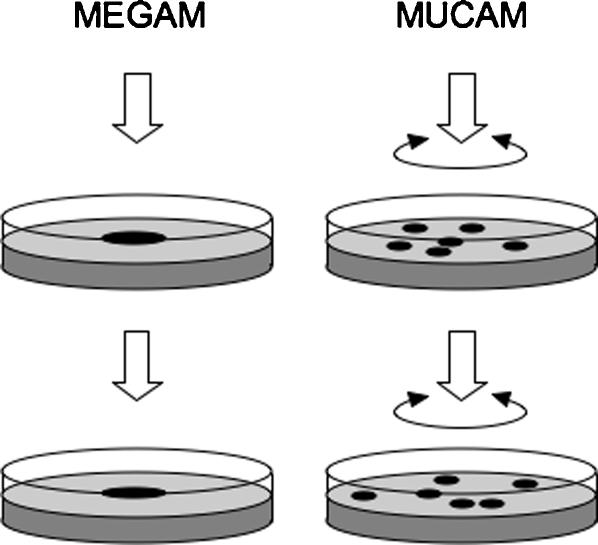
The agar plate can be used as mega-colony or multiple-colony agar microcosms (MEGAM/MUCAM) in which bacterial adaptation and colony interactions can be observed. In the MEGAM microcosm, a single mixed colony is incubated in the centre of the plate. The colony is harvested and an appropriate dilution is used to inoculate a second plate, again as a single colony. In the MUCAM microcosm, the inoculation is spread across the surface of the plate to allow the development of multiple colonies with differing degrees of separation and overlap. The colonies are pooled together on harvest to produce the inoculation for the subsequent plate, which again is spread across the surface of the entire plate.

**Figure 5:**
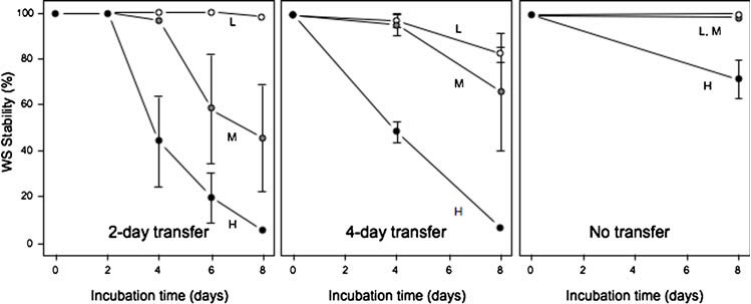
The WS genotype was maintained on agar plates at low population densities in the mega-colony agar microcosm (MEGAM) experiment, but was rapidly lost at high population densities. The mega-colony was recovered from plates every 2 or 4-days and used to inoculate fresh plates with a single 5 µl drop (a third set of populations was incubated for 8-days without transfer). The percentage of cells still showing the WS phenotype (WS Stability) after each period is indicated. Populations were transferred at three densities, high (H) (∼10^7^ *cfu* per drop), medium (M) (∼10^4^ *cfu* per drop), and low (L) (∼10 *cfu* per drop). Colonies were incubated on KB plates at 28°C before transfer to new plates. Mean±SE indicated.

WS reversion to SM-LR and adaptation to more stable WS-like genotypes was investigated using two applications of the two-dimensional agar plate microcosm, MEGCAM and MUCAM (**Figure 4**). In the first, bacterial populations were grown as a single large, mixed colony resulting from inoculation with a 5 µl droplet in the centre of each KB plate (MEGAM – mega-colony agar microcosm). In the second procedure, the inoculation was spread across the entire agar surface resulting in multiple individual colonies overlapping to different degrees (MUCAM – multi-colony agar microcosm). In each protocol, colony material was scraped and the bacteria resuspended to allow inoculation of fresh plates in a transfer regime and/or to enumerate WS numbers. In the test of WS stability and persistence in the MEGAM microcosm inoculated with WS, cells were transferred at three different cell densities following a 2 or 4-day transfer regime, or left on the original plates for the full 8-days of the experiment (8-days represented between ∼10 (high-density regime, 8-day incubation) – 120 (low-density regime, 2-day transfers) average minimal number of generations as calculated from the raw *cfu* data) (**Figure 5**). WS stability between low, medium and high-density colonies differed significantly after 8-days incubation without transfer (ANOVA, *F*_2,6_ = 10.7673, *P* = 0.0103), with the lowest stability seen for colonies with the highest cell density. Similarly, WS stability differed significantly after two 4-day transfers (ANOVA, *F*_2,6_ = 7.0142, *P* = 0.0269) and after four 2-day transfers (ANOVA, *F*_2,6_ = 11.4510, *P* = 0.0089), again showing the lowest stability in colonies with the highest cell density.

**Figure 6:**
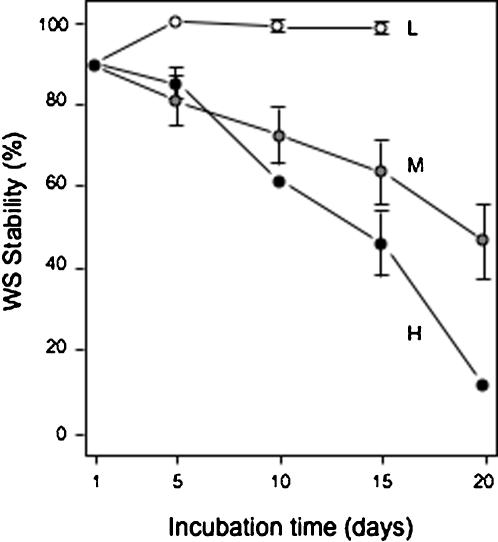
The WS genotype was maintained on agar plates at low population densities in the multiple-colony agar microcosm (MUCAM), but was rapidly lost at high population densities. Colonies were recovered from plates every 5-days as a single population and used to inoculate fresh plates. The percentage of cells still showing the WS phenotype (WS Stability) after each 5-day period is indicated. Populations were transferred at three densities, high (H) (1,000–2,000 *cfu* per plate), medium (M) (100–200 *cfu* per plate), and low (L) (10–20 *cfu* per plate). Colonies were incubated on KB plates at 28°C before transfer to new plates. Mean±SE indicated.

In the MUCAM microcosm inoculated with WS, populations were transferred every 5-days for a total of 20-days, and plating densities chosen such that low-density plates contained 10–20 separate colonies, medium-density plates contained 100–200 colonies with some overlap, and high-density plates almost confluent growth after 2-days of incubation (20-days represented between ∼93 (high-density regime) – 120 (low-density regime) average minimal number of generations as calculated from the raw *cfu* data) (**Figure 6**). By day 15 after only three transfers, there was a significant difference in WS stability (ANOVA, *F*_2,5_ = 11.3950, *P* = 0.0137). Although MEGAM/MUCAM microcosms provide different environments for WS and SM-LR competition and adaptation, the results were broadly similar. WS stability was highest when cells were transferred at lower population densities and when populations were incubated on plates for longer periods between transfers. This suggests that the rapid surface colonisation ability of the WS was an advantage under these conditions, but not under higher population densities and shorter transfer regimes where higher growth rates (and colony expansion upwards rather than across) were favoured.

### Evolution of the Hardy WS isolates in the two-dimensional agar plate microcosm

**Figure 7:**
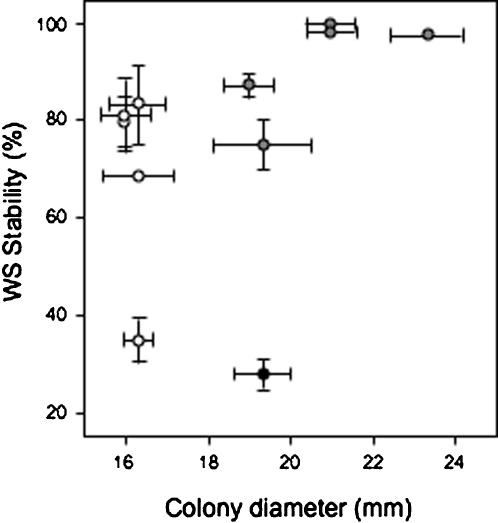
Plate-adapted Hardy WS isolates from the MUCAM experiment show a lowered degree of WS reversion, enhanced WS stability and different colony sizes compared to the ancestral WS strain. The percentage of cells still showing the WS phenotype (WS Stability) recovered from colonies is graphed against the colony diameter for each isolate obtained from high (white circles) and medium-density (grey circles) evolved populations, plus the WS (black circle). Colonies were incubated on KB plates at 28°C for 3-days before sampling. Mean±SE indicated.

Although in both the MEGAM/MUCAM microcosms mutations occurred in the WS population to produce SM-LR, mutations might also be expected to result in better-adapted lineages still retaining the WS phenotype. This was confirmed by analysing 10 ‘Hardy WS’ isolates obtained from H and M MUCAM plates after a total of 22-days adaptation (corresponding to ∼100 generations). Significant changes in both WS stability and colony diameters were found between the two groups of Hardy WS isolates and the ‘ancestral’ WS (**Figure 7**), indicating that during the incubation on MUCAM plates the WS maladaptation had been reduced or partially compensated in these particular WS-like lineages (WS stability, ANOVA, *F*_2,28_ = 17.4762, *P*<0.0001; Colony diameter, ANOVA, *F*_2,30_ = 34.4519, *P*<0.0001). However, no significant difference between the two groups and the WS were seen with Congo Red-binding, a measure of both cellulose and attachment factor expression (ANOVA, *F*_2,52_ = 1.7700, *P* = 0.1804). Within the Hardy WS isolates, only WS stability and colony diameter were correlated (Pearson's correlation, *r* = 0.36676, *P* = 0.0424) (all other pair-wise correlations between stability, diameter and Congo Red-binding were not significant). However, whereas three of the five Hardy WS isolates from the M population showed an increased average colony size, all of the H population isolates showed reduced colony sizes. This difference suggests that under high population density conditions, the WS appear to evolve towards an SM-like strategy (i.e. less outward growth of the colony), whereas under medium population density the WS evolve an enhanced WS-like strategy (i.e. greater outward growth), highlighting the very different selective pressures of these environments.

### Plate-adaptation of the WS is an example of the constant evolution of bacterial populations

The obvious instability of the WS genotype and rapid appearance of SM-LR in developing WS colonies suggested that the WS was maladapted to agar plates, in stark contrast to the ecological success of this genotype in static liquid microcosms. However, extended propagation on plates over a relatively short period of ∼100 generations resulted in WS lineages showing greater levels of stability. Whilst experimental bacterial populations are often propagated over thousands of generations, changes in colony morphology and fitness have been observed within far shorter intervals, including populations propagated on agar surfaces [**27**]. The original diversification of *P. fluorescens* SBW25 giving rise to the WS genotype was observable within 30 generations (estimated from *cfu* data in [**1**]). The WS genotype is not static either, as WS genotypes are known to accumulate measurable metabolic defects and reduced fitness within 100 generations, whilst compensatory adaptation (mutations which lead to an increase in fitness) in WS lineages has been seen within a similar interval in liquid microcosms [**25**]. The opportunities offered by novel and under-populated environments present significant selective pressure to bacterial populations, where relatively minor changes in genotype can result in better-adapted phenotypes allowing rapid colonisation and more effective competition for limited resources. The trade-off between colonisation and competitive ability affected by environmental conditions observed in the adaptation of the WS to agar plates has relevance to the intermediate dispersal hypothesis, optimal foraging, and the evolution of dispersal in general [**31]**, [32], [**33**]. The adaptation of the WS to the two-dimensional agar plate microcosm is yet another example of the constant adaptation and response of bacterial populations to new environments and opportunities.

## Materials and Methods

The *Pseudomonas fluorescens* strains used in this work are derivatives of the wild-type *P. fluorescens* SBW25 (also referred to as ‘smooth’ (SM) or ‘ancestral’) [**8**]. The Wrinkly spreader (WS) was a biofilm-forming strain obtained from the SM (SM *wspF*S301R) [**2]**, [**19**]. WS-4 is WS *wspR*::mini-Tn*5*; WS-12 is WS *pgi*::mini-Tn*5*, and WS-13 is WS *wssB*::mini-Tn*5* [**2**]. pCSA expresses the complete *wss* operon [**2**], and pVSP61-*wspR19-ΩTc^R^* the constitutively-active WspR19 (R129C) mutant protein [**28**]. Both were transferred by conjugation from *Escherichia coli* hosts into SBW25 [**2**] and maintained with 12.5 µg.ml^−1^ tetracycline. Bacteria were cultured in King's B (KB) [**5**], Lauria-Bertani broth (LB) [**6**] or M9 minimal media [**29**] (agar was added to 1.5% w/v for all plates) and grown at 28°C unless otherwise stated.

Cell numbers were determined from colony forming units (*cfu*) and growth rates reported as *cfu*_initial_/*cfu*_final_ relative to the WS. The average number of generations (log_2_[*f*/*i*]) and WS competitive fitness (W) relative to SM (*ln*[WS*_f_*/WS*_i_*]/*ln*[SM*_f_*/SM*_i_*]) [**30**] were calculated directly from *cfu* data, where *i* and *f* were initial and final population sizes, respectively. The reversion frequency of WS to SM-like phenotypes was determined by incubating colonies before determining the percentage of WS and SM-like cells. Briefly, a plate was inoculated with a 5 µl drop of culture, air-dried then incubated at the appropriate temperature. At the time of harvest, the entire colony was recovered and resuspended in KB. Appropriate dilutions were made and plated onto fresh plates. After incubation at 28°C for 48 hr, colony phenotypes were assessed to determine the percentage of cells retaining the WS phenotype, reported as WS Stability (%). Assays were performed with 2–5 independent replicates and mean±standard errors (SE) provided where necessary. Data were examined using JMP Statistical Discovery Software (SAS) and the VassarStats web site for Statistical Computation.

Cellulose expression was determined quantitatively by Congo Red (SIGMA)-binding and qualitatively by fluorescent microscopy after staining with Calcofluor (SIGMA Fluorescent Whitener 28) [**3**]. Complete *wspF* sequences were obtained by PCR-based automated DNA sequencing [**19**] and compared to the WS *wspF*S301R sequence (the wild-type WspF sequence is AA092338) using Sequence Navigator (PE Applied Biosystems).
